# Neonatal atrial flutter after insertion of an intracardiac umbilical venous catheter

**DOI:** 10.1016/j.rppede.2015.10.002

**Published:** 2016

**Authors:** Marcos Moura de Almeida, Wládia Gislaynne de Sousa Tavares, Maria Mônica Alencar Araripe Furtado, Maria Marcia Farias Trajano Fontenele

**Affiliations:** Maternidade Escola Assis Chateaubriand, Universidade Federal do Ceará (UFC), Fortaleza, Ceará, Brazil

**Keywords:** Atrial flutter, Catheters, Newborn

## Abstract

**Objective:**

To describe a case of neonatal atrial flutter after the insertion of an intracardiac umbilical venous catheter, reporting the clinical presentation and reviewing the literature on this subject.

**Case description:**

A late-preterm newborn, born at 35 weeks of gestational age to a diabetic mother and large for gestational age, with respiratory distress and rule-out sepsis, required an umbilical venous access. After the insertion of the umbilical venous catheter, the patient presented with tachycardia. Chest radiography showed that the catheter was placed in the position that corresponds to the left atrium, and traction was applied. The patient persisted with tachycardia, and an electrocardiogram showed atrial flutter. As the patient was hemodynamically unstable, electric cardioversion was successfully applied.

**Comments:**

The association between atrial arrhythmias and misplaced umbilical catheters has been described in the literature, but in this case, it is noteworthy that the patient was an infant born to a diabetic mother, which consists in another risk factor for heart arrhythmias. Isolated atrial flutter is a rare tachyarrhythmia in the neonatal period and its identification is essential to establish early treatment and prevent systemic complications and even death.

## Introduction

Atrial flutter is a rare arrhythmia in the neonatal period. Its low incidence makes it difficult to carry out studies and justifies the small number of publications about the best treatment and long-term prognosis.^1^^,^ 2

Umbilical catheterization is commonly used in neonatal management for the administration of parenteral nutrition, hypertonic solutions, blood products, blood pressure monitoring and medication infusion. This procedure, although easy to perform, has potential risks, including catheter-related infection, thrombosis, myocardial perforation, pleural and pericardial effusions and arrhythmias.^3^ Catheters should ideally be positioned between the inferior vena cava and the right atrium. Catheters that go beyond the right atrium can get lodged in the superior vena cava, right ventricle, but usually pass through the foramen ovale and become lodged in the left atrium, which can lead to endocardial injury.^4^ The association between umbilical venous catheterization and cardiac arrhythmias is mainly reported when the catheter is misplaced, in an intracardiac position.^4^^–^ 8

Abnormalities in the fetal heart rate occur in 2% of pregnancies.^9^ Fetuses of diabetic mothers require special care, both in the prenatal and early neonatal periods. These newborns are usually large for gestational age (LGA), have higher admission rates at neonatal intensive care units (NICUs) and higher mortality rates than newborns who are adequate for gestational age, as well as a higher frequency of atrial arrhythmia.^9^^,^ 10

The aim of this article is to report a case of an LGA newborn, born to a diabetic mother, who developed atrial flutter after the placement of an intracardiac umbilical venous catheter, reporting the clinical outcome and performing a brief literature review on the topic.

## Case description

The patient was an infant born to a diabetic mother with gestational hypertension and urinary tract infection, of which treatment was started during labor. The patient was born by cesarean section due to obstetric indication at 35 weeks of gestational age, according to the last menstrual period, with Apgar scores of 3 and 8 in the first and fifth minutes of life, respectively, and birth weight of 3755g being classified as LGA according to Alexander's curve of reference values of neonatal weight.^11^

The patient had early mild respiratory distress, with no other alterations in the physical examination and asymptomatic hypoglycemia in the first hour of life, resolved after formula administration. The newborn was referred to the medium-risk neonatal unit using oxygen with inspiratory oxygen fraction of 40%. Ten hours after birth, the newborn showed worsened respiratory distress and was admitted at the neonatal ICU for ventilatory support with continuous positive airway pressure (CPAP) and early antibiotic therapy for rule-out sepsis. Umbilical venous catheterization was performed approximately 12 h after birth due to the difficulty in obtaining peripheral venous access.

**Figure 1:**
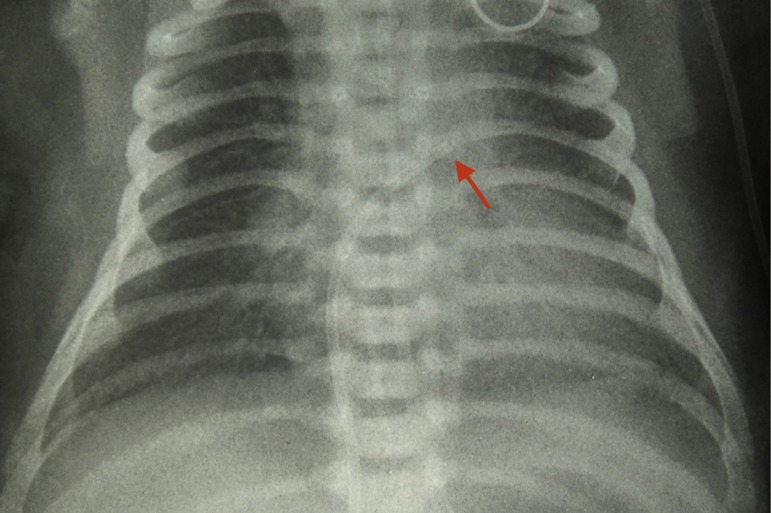
Intracardiac umbilical venous catheter in the left atrial region.

Soon after the procedure, the patient showed persistent tachycardia (190–230 beats per minute) and worsening of respiratory pattern, requiring tracheal intubation. Chest radiography showed normal cardiac area, clear lungs and intracardiac umbilical catheter in the left atrium region (Fig. 1), which was repositioned. The patient, however, persisted with tachycardia. An electrocardiogram was then performed, which confirmed the supraventricular tachycardia, suggestive of atrial flutter.

**Figure 2:**
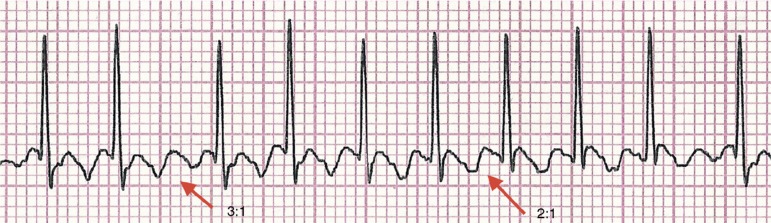
Electrocardiogram showing the “sawtooth” or “picket fence” pattern of atrial flutter, with 3:1 and 2:1 atrioventricular conduction in the D2 lead.

The diagnosis was confirmed after adenosine administration (50mcg/kg/dose), when the typical “sawtooth” or “picket fence” pattern was observed in the P wave, characteristic of atrial flutter, with 460 atrial contractions per minute (Fig. 2). The infant developed hemodynamic instability, weak pulses and slowed peripheral perfusion, thus being submitted to electrical cardioversion (0.5J/kg), with sinus rhythm return. Administration of amiodarone (5mg/kg) was initiated, and the newborn progressed with no new tachyarrhythmias, maintaining hemodynamic stability. The echocardiography performed on the day after cardioversion showed mild pulmonary hypertension and a 2.6-mm patent foramen ovale.

The patient remained stable, with progressive improvement of respiratory distress, and was extubated after four days, being discharged from the neonatal ICU at eight days old, asymptomatic.

## Discussion

Atrial flutter is the most commonly reported tachyarrhythmia in the fetal period, being rare in its isolated form in the neonatal period.^12^ Its etiology is uncertain, but there is an association with structural heart diseases, which should be promptly ruled out by echocardiography. The presence of structural alterations is correlated with worse prognosis.^2^

The diagnosis is often simple, with the electrocardiogram showing the typical “sawtooth” or “picket fence” pattern, better observed in leads II, III and aVF. The clinical presentation of atrial flutter depends on the ventricular response to atrial tachyarrhythmia. In newborns, the atrial rate is 400 beats per minute (bpm), with an atrioventricular conduction ratio of 2:1, resulting in a ventricular rate of approximately 200bpm.^12^ The patient described had a ventricular rate of around 190–230bpm and atrial rate of 380–460bpm.

Complications related to the umbilical venous catheterization are reported regarding its inadequate positioning.^4^^–^ 8 There are methods to determine the correct insertion length of umbilical venous catheters.^13^^,^ 14 One approach uses equations based on the newborn's weight, as described by Shukla et al.^13^ Dunn's method, the most commonly used one, is based on the measurement of the shoulder-navel distance. This method is hindered by numerous important limitations, including interpersonal variations.^14^ It is not known whether the estimated insertion length based on these methods is accurate.^15^ Once the patient is catheterized, the location is routinely verified by a chest X-ray. In chest X-rays in the anteroposterior view, the ideal position for the catheter tip is located between the T8 and T9 thoracic vertebrae. In this position, 90% of the catheters will have their distal end placed between the right atrium and the inferior vena cava.

It is noteworthy that 56% of umbilical venous catheters, when radiography was used to verify the location of the catheter tip, had to be repositioned because they were placed in an unsatisfactory site.^16^^,^ 17 When comparing the methods to identify the correct position of catheters, ultrasound is a more accurate complementary method of examination than chest X-ray to determine the route and the position of venous umbilical catheters.^16^^,^ 17 The first difficulty is to cross the ductus venosus and then reach its center position. This difficulty justifies the complications related to poor positioning.^16^ In the patient described in this report, the Dunn method was used as a reference for catheter positioning, with its position being assessed by chest X-ray, according to the service routine.

The association between diabetic mothers and newborns with atrial arrhythmias has been reported in the literature. Some studies have shown that fetuses and neonates with atrial tachyarrhythmias are most commonly large for gestational age or the offspring of diabetic mothers, as the patient shown here. They have cardiac function alterations, regardless of the presence of ventricular hypertrophy. There is the hypothesis that diastolic dysfunction, with subsequent atrial dilation, may predispose infants of diabetic mothers to atrial arrhythmias.^9^

The management of newborns with cardiac arrhythmias secondary to umbilical venous catheterization has not been systematically studied. As arrhythmias often occur due to poor positioning of the catheter, the first step should be to pull it back or even remove the catheter. However, in our case, catheter pullback was not effective. Treatment to bring the heart back to sinus rhythm can be pharmacological (antiarrhythmics such as adenosine, digoxin, amiodarone, etc.) or electric (cardioversion or transesophageal atrial stimulation). Adenosine administration may be effective, but it does not always treat tachycardia of atrial origin, such as atrial flutter, as seen in the patient described here.^18^^–^ 20 However, adenosine administration can help identify atrial flutter, as it produces a transient atrioventricular block, demonstrating the arrhythmia-characteristic wave in the ECG.^18^^,^ 19 New drugs, most of which have already been used in adults and children, are being studied, such as ibutilide and propafenone, and there have been reports of their use in newborns.^21^^,^ 22 When there is no response to pharmacological treatment, therapy may require synchronized cardioversion or transesophageal atrial pacing, with high probability of sinus rhythm conversion.^1^^,^ 21 ^–^ 24 In cases with hemodynamic instability, electrical cardioversion should be preferably used, as in the case reported here.

Leroy et al.^5^ described a similar case, in which a full-term newborn developed atrial flutter after an umbilical venous catheter was placed in the left atrium. Treatment consisted in repositioning the catheter and transesophageal atrial pacing, with good evolution. Sinha et al.^7^ also reported a patient with hemodynamic instability due to atrial flutter after catheterization, resolved after synchronized cardioversion.

Fetal and neonatal atrial flutter is associated with significant morbidity. However, mortality seems to be more often related to the presence of associated medical conditions.^25^ Pharmacological or electrical cardioversion to normal sinus rhythm may be effective and, once such reversal is achieved, the patient does not seem to have a risk of recurrence of atrial flutter, except when there is an accessory pathway; in this case, supraventricular tachycardia episodes may occur. In patients without an accessory pathway, it is not usually necessary to maintain the long-term medication.^25^

Considering the small amount of data available in the national and international literature, mainly from the isolated case reports, there is scarce information about the precise association between neonatal arrhythmias and the umbilical catheterization, including the actual incidence of atrial flutter and the indication of a “universal” therapeutic approach for it. The association between cardiac arrhythmias and maternal diabetes is a research field yet to be developed, which can contribute to the prevention of this disease, supported by a better understanding of the physiopathological mechanisms of these entities.
