# Supplementary material for: Neonatal atrial flutter after insertion of an intracardiac umbilical venous catheter
Source: Rev Paul Pediatr. 2016 Jan-Mar;34(1):132–5. doi: 10.1016/j.rppede.2015.10.002 (PMC4795732; doi:10.1016/j.rppede.2015.10.002)
Supplement: Translation

# Translation: *Flutter* atrial neonatal após inserção de cateter umbilical intracardíaco

**Authors:** Marcos Moura de Almeida, Wládia Gislaynne de Sousa Tavares, Maria Mônica Alencar Araripe Furtado, Maria Marcia Farias Trajano Fontenele

**Affiliations:** Maternidade Escola Assis Chateaubriand, Universidade Federal do Ceará (UFC), Fortaleza, Ceará, Brasil

**Keywords:** Flutter atrial, Cateteres, Recém-nascido

## Abstract

**Objetivo:**

Descrever um caso de *flutter* atrial neonatal após a inserção de um cateter venoso umbilical intracardíaco, relatar sua evolução clínica e fazer uma revisão bibliográfica sobre o tema.

**Descrição do caso:**

Recém-nascido pré-termo tardio de 35 semanas de idade gestacional, filho de mãe diabética, grande para a idade gestacional, com desconforto respiratório precoce e risco para infecção neonatal, que necessitou de cateterização venosa umbilical. Após o procedimento, o paciente apresentou taquicardia. A radiografia torácica evidenciou posição intracardíaca inadequada do cateter umbilical, que foi tracionado, e o neonato permaneceu taquicárdico. O eletrocardiograma permitiu o diagnóstico de *flutter* atrial. Por conta da instabilidade hemodinâmica foi feita cardioversão elétrica, com sucesso.

**Comentários:**

A relação entre arritmias atriais e cateteres umbilicais mal posicionados tem sido descrita na literatura, mas, nesse caso, vale ressaltar o fato de o paciente ser filho de mãe diabética, o que consiste em outro fator de risco para as arritmias cardíacas. O *flutter* atrial isolado é uma taquiarritmia rara no período neonatal. O seu reconhecimento é fundamental para um tratamento precoce e para evitar complicações sistêmicas e até mesmo fatais.

## Introdução

O *flutter* atrial é uma arritmia incomum no período neonatal. A sua baixa incidência dificulta a feitura de estudos e justifica as poucas publicações sobre a melhor conduta terapêutica e o prognóstico em longo prazo.^1^^,^ 2

O cateterismo umbilical é comumente usado no manejo neonatal para administração de nutrição parenteral, soluções hipertônicas, hemoderivados, monitoração da pressão arterial e infusão de medicações. Este procedimento, apesar de fácil execução, tem riscos potenciais, incluindo infecção relacionada ao cateter, tromboses, perfuração miocárdica, efusões pleural e pericárdica e arritmias.^3^ Os cateteres devem ser idealmente posicionados entre a veia cava inferior e o átrio direito. Cateteres que ultrapassam o átrio direito podem se alojar na veia cava superior, ventrículo direito, mas geralmente passam através do forame oval e se alojam no átrio esquerdo, o que podendo levar a lesão no endocárdio.^4^ A associação entre cateterismo venoso umbilical e arritmias cardíacas é relatada principalmente quando o cateter está mal

posicionado, intracardíaco.^4^^-^ 8

Anormalidades no ritmo cardíaco fetal ocorrem em 2% das gestações.^9^ Fetos de mães diabéticas requerem cuidado especial, tanto no período pré-natal como no neonatal precoce. Esses recém-nascidos são geralmente grandes para a idade gestacional (GIG), têm maiores taxas de admissão em unidades de terapia intensiva (UTI) neonatal e maior mortalidade do que recém-nascidos adequados para idade gestacional, além de maior frequência de arritmia atrial.^9^^,^ 10

O objetivo deste artigo é relatar um caso de recém-nascido GIG e filho de mãe diabética que desenvolveu *flutter* atrial após a inserção de cateter venoso umbilical intracardíaco, relatar sua evolução clínica e fazer uma breve revisão bibliográfica sobre o tema.

## Descrição do caso

Recém-nascido filho de mãe diabética, com doença hipertensiva específica da gravidez e infecção do trato urinário cujo tratamento foi iniciado durante o trabalho de parto. O paciente nasceu por parto cesáreo por indicação obstétrica com 35 semanas de idade gestacional, de acordo com a data da última menstruação, com boletim de Apgar no primeiro e quinto minuto de vida de, respectivamente, três e oito, peso de nascimento de 3.755g, classificado como GIG pela curva de valores de referência de peso neonatal de Alexander.^11^

Apresentou desconforto respiratório precoce leve, sem demais alterações no exame físico, e hipoglicemia assintomática na primeira hora de vida, resolvida após administração de dieta. O recém-nascido foi levado para a unidade neonatal de médio risco em uso de oxigênio com fração inspiratória de 40%. Com 10 horas de vida apresentou pioria do desconforto respiratório e foi levado à UTI neonatal para suporte ventilatório em dispositivo de pressão positiva contínua de vias aéreas (CPAP) e início de antibioticoterapia pelo risco de infecção neonatal. Foi feito cateterismo umbilical venoso com aproximadamente 12 horas de vida por dificuldade na obtenção de acesso venoso periférico.

Logo após o procedimento, o paciente apresentou taquicardia persistente (190-230 batimentos por minuto) e pioria do padrão respiratório. Foi necessária a intubação orotraqueal. A radiografia de tórax evidenciou área cardíaca normal, campos pulmonares limpos e cateter umbilical intracardíaco, em topografia do átrio esquerdo (fig. 1), o qual foi reposicionado. O paciente, porém, manteve-se taquicárdico. Foi, então, feito o eletrocardiograma, que confirmou uma taquicardia supraventricular, sugestiva de *flutter* atrial.

**Figura 1:** Cateter umbilical venoso intracardíaco em topografia de átrio esquerdo.

O diagnóstico foi confirmado após a administração de adenosina (50mcg/kg/dose), quando foi evidenciado o aspecto serrilhado da onda P característico do *flutter* atrial, com 460 contrações atriais por minuto (fig. 2). O neonato evoluiu com instabilidade hemodinâmica, pulsos fracos e perfusão periférica lentificada. Foi feita cardioversão elétrica (0,5 J/kg) com retorno ao ritmo sinusal. Iniciada a administração de amiodarona (5mg/kg) e o recém-nascido evoluiu sem novas taquiarritmia, manteve a estabilidade hemodinâmica. O ecocardiograma no dia seguinte à cardioversão evidenciou hipertensão pulmonar leve e forame oval pérvio 2,6mm.

**Figura 2:** Eletrocardiograma que evidencia o padrão serrilhado do *flutter* atrial com condução atrioventricular 3:1 e 2:1 na derivação D2.

O paciente permaneceu estável, com melhoria progressiva do desconforto respiratório, foi extubado após quatro dias e recebeu alta da UTI neonatal com oito dias de vida, assintomático.

## Discussão

O *flutter* atrial é a taquiarritmia mais comumente relatada no período fetal, é rara, de forma isolada, no período neonatal.^12^ A etiologia é incerta, mas há relação com cardiopatias estruturais, que devem ser prontamente afastadas pelo ecocardiograma. A presença de alterações estruturais correlaciona-se a um pior prognóstico.^2^

O diagnóstico muitas vezes é simples, com eletrocardiograma que evidencia ondas típicas em dente de serra, mais bem evidenciadas nas derivações II, III e aVF. A apresentação clínica do *flutter* atrial depende da resposta ventricular à taquiarritmia atrial. No recém-nascido, a frequência atrial é de cerca de 400 batimentos por minuto (bpm), com condução atrioventricular na proporção de 2:1, resulta em uma frequência ventricular de aproximadamente 200bpm.^12^ O paciente aqui descrito apresentou frequência ventricular em torno de 190-230bpm e atrial de 380-460bpm.

Complicações relacionadas ao cateterismo umbilical venoso são relatadas quanto ao seu posicionamento inadequado.^4^^-^ 8 Existem métodos para determinar o comprimento correto de inserção de cateteres venosos umbilicais.^13^^,^ 14 Um deles usa equações com base no peso do recém-nascido, conforme descrito por Shukla *et al*.^13^ O método de Dunn, mais comumente aplicado, baseia-se na medição da distância ombro-umbigo. Esse método é dificultado por várias limitações importantes, incluindo variações interpessoais.^14^ Não é conhecido se a estimativa do comprimento de inserção com base nesses métodos é precisa.^15^ Uma vez cateterizado o paciente, a localização é feita de forma rotineira por radiografia de tórax. Na radiografia de tórax em incidência anteroposterior a posição ideal da ponta do cateter se situa entre as vértebras torácicas T8-T9. Nessa posição, 90% dos cateteres estarão com sua extremidade distal entre o átrio direito e a veia cava inferior.

Ressalta-se que 56% dos cateteres venosos umbilicais, quando se usou a radiografia como método para verificar a localização da ponta do cateter, precisaram ser reposicionados por estar em local não satisfatório.^16^^,^ 17 Comparando os métodos para identificar a posição correta dos catéteres, a ultrassonografia é um exame complementar mais acurado do que a radiografia torácica para determinar a rota e a posição de cateteres umbilicais venosos.^16^^,^ 17 A primeira dificuldade é atravessar o duto venoso e, então, atingir sua posição central. Essa dificuldade justifica as complicações relacionadas ao seu mau posicionamento.^16^ No paciente descrito neste relato, foi usado o método de Dunn como referência para posicionamento do cateter e avaliada a posição por radiografia de tórax, de acordo com a rotina do serviço.

A relação entre mães diabéticas e neonatos com arritmias atriais tem sido relatada na literatura. Alguns já demonstraram que fetos e neonatos com taquiarritmias atriais são mais comumente grandes para idade gestacional ou filhos de mãe diabética, como o paciente aqui apresentado. Esses têm alteração da função cardíaca, independentemente da presença de hipertrofia ventricular. Existe a hipótese de que a disfunção diastólica, com subsequente dilatação atrial, possa predispor os neonatos de mães diabéticas a arritmias atriais.^9^

O manejo dos recém-nascidos com arritmias cardíacas secundárias à cateterização venosa umbilical não foi estudado de modo sistemático. Como as arritmias ocorrem geralmente por mau posicionamento do cateter, o primeiro passo deve ser tracionar ou até mesmo remover o cateter. No entanto, em nosso caso, a tração não foi resolutiva. O tratamento para que o ritmo cardíaco volte a ser o sinusal pode ser farmacológico (antiarrítmicos como adenosina, digoxina, amiodarona etc.) ou elétrico (cardioversão ou estimulação atrial transesofágica). A administração de adenosina pode ser eficaz, mas nem sempre trata as taquicardias de origem atrial, como o *flutter* atrial, conforme observado no paciente aqui descrito.^18^^-^ 20 No entanto, a administração de adenosina pode ajudar a identificar o *flutter* atrial, pois essa produz um bloqueio atrioventricular transitório e evidencia, no eletrocardiograma, a onda característica da arritmia.^18^^,^ 19 Novas drogas, a maioria já usada em adultos e crianças maiores, estão sendo estudadas, como a ibutilida e a propafenona. Há relatos de casos de seu uso em recém-nascidos.^21^^,^ 22 Quando não há resposta ao tratamento farmacológico, a terapêutica pode requerer a cardioversão sincronizada ou a estimulação atrial transesofágica, com grande probabilidade de conversão ao ritmo sinusal.^1^^,^ 21 ^-^ 24 Nos casos com instabilidade hemodinâmica, a cardioversão elétrica deve ser usada preferencialmente, como ocorreu no caso aqui relatado.

Leroy *et al*.^5^ descreveram um caso semelhante, no qual um recém-nascido a termo evoluiu com *flutter* atrial após inserção de cateter umbilical venoso posicionado em átrio esquerdo. O tratamento estabelecido foi o reposicionamento do cateter e estimulação atrial transesofágica, com boa evolução. Sinha *et al*.^7^ também relataram um paciente com instabilidade hemodinâmica por *flutter* atrial após cateterismo, resolvido após cardioversão sincronizada.

O *flutter* atrial fetal e neonatal é associado à morbidade significativa. No entanto, a mortalidade parece estar mais relacionada à presença de condições médicas associadas.^25^ A cardioversão farmacológica ou elétrica para o ritmo sinusal pode ser eficaz e uma vez que tal reversão é atingida, o paciente parece não apresentar risco de recorrência do *flutter* atrial, exceto quando existe uma via acessória. Nesse caso, episódios de taquicardia supraventricular podem ocorrer. Em pacientes sem uma via acessória. geralmente não é necessário manter a medicação em longo prazo.^25^

Pelo pequeno conjunto de dados disponíveis na literatura nacional e internacional, oriundos principalmente de relatos de caso isolados, é deficiente o conhecimento quanto à relação exata entre arritmias neonatais e o cateterismo umbilical, incluindo a incidência real do *flutter* atrial e a indicação de uma abordagem terapêutica universal para o mesmo. A relação entre arritmias cardíacas e diabete materna é um campo de pesquisa a ser desenvolvido, que poderá contribuir para a prevenção dessa enfermidade, apoiada numa maior compreensão dos mecanismos fisiopatológicos dessas entidades.
